# Supplementary material for: Cancer-derived small extracellular vesicles promote angiogenesis by heparin-bound, bevacizumab-insensitive VEGF, independent of vesicle uptake
Source: Commun Biol. 2019 Oct 18;2:386. doi: 10.1038/s42003-019-0609-x (PMC6802217; doi:10.1038/s42003-019-0609-x)
Supplement: Supplementary file 1 — Supplementary Figures [file 42003_2019_609_MOESM1_ESM.pdf]

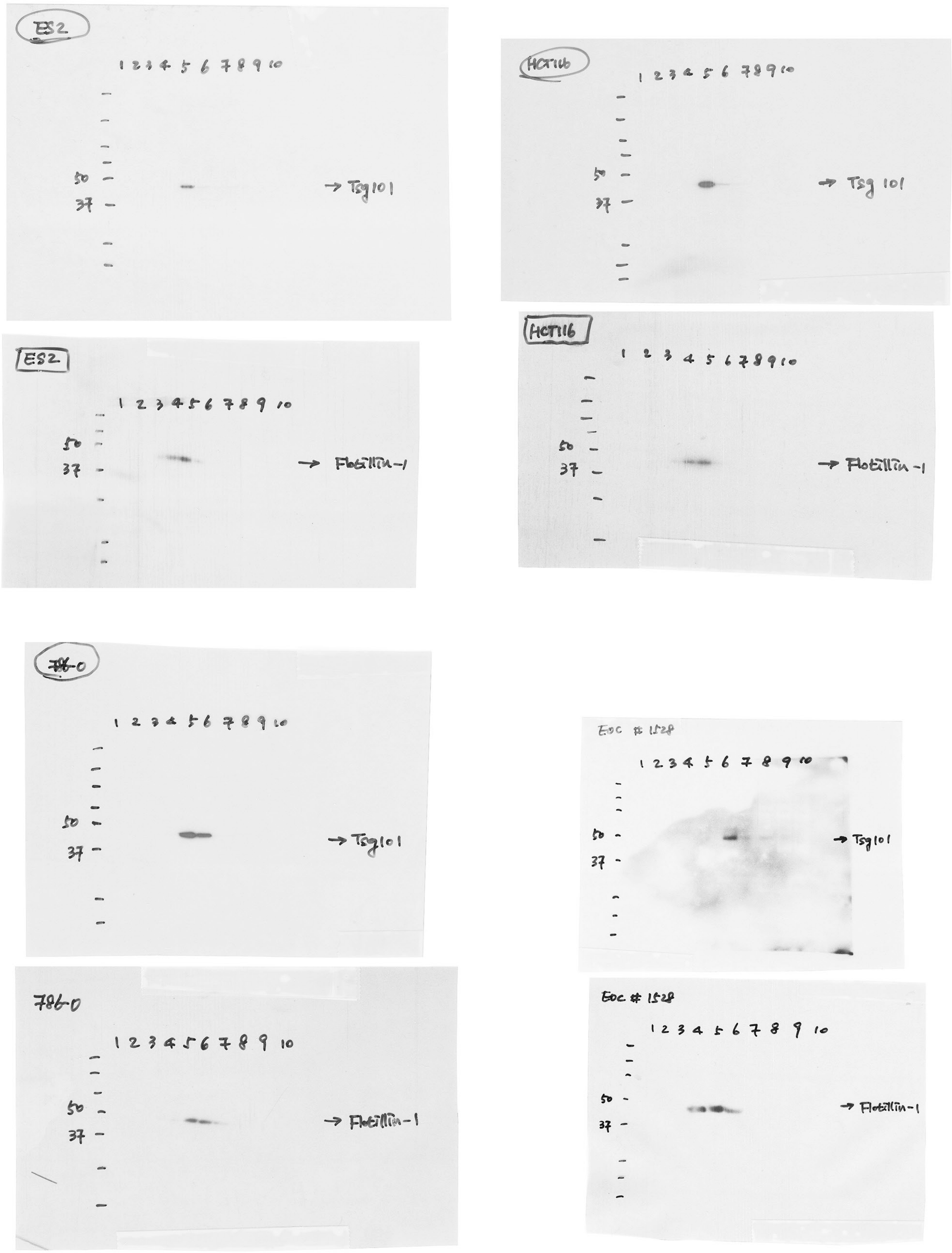

Supplementary Figure 1. Uncropped immunoblots of TSG101 and flotillin-1 shown in Fig. 1a.

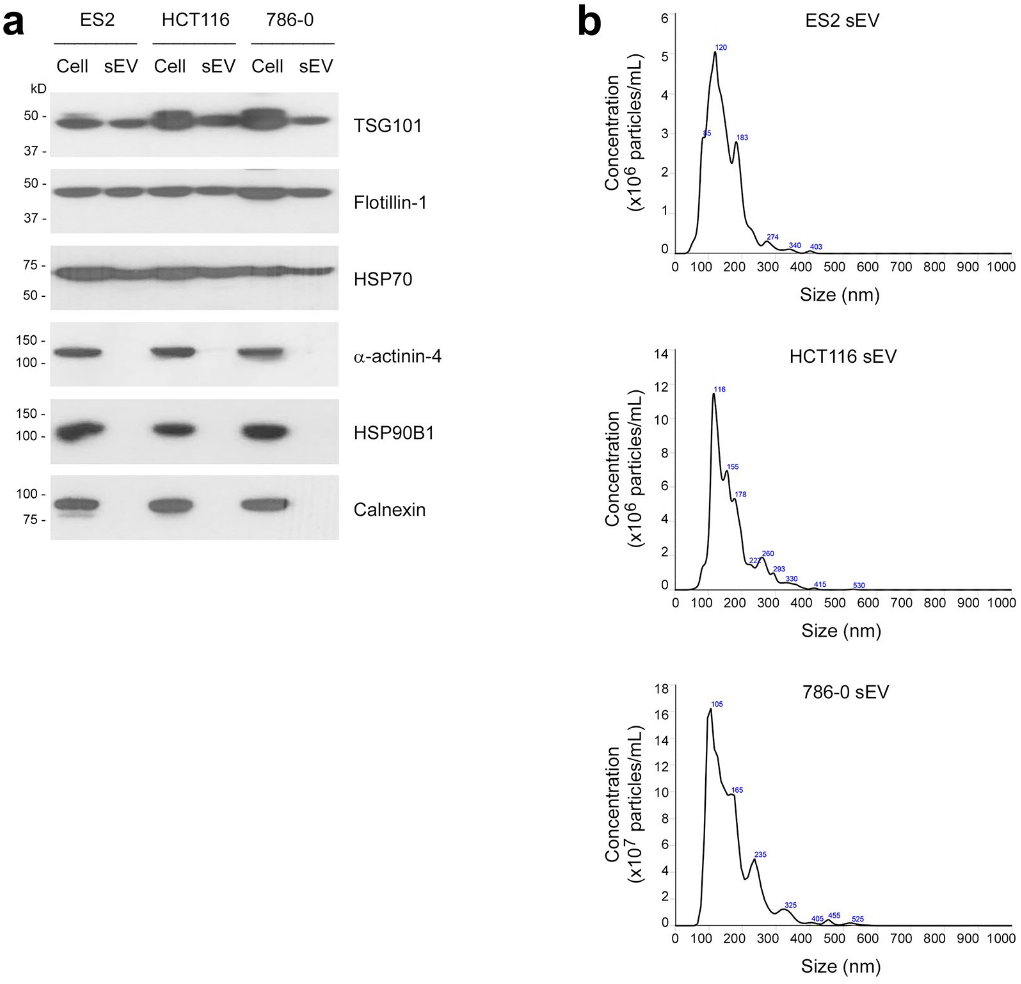

Supplementary Figure 2. Analysis of markers and particle size distribution of sEVs secreted by cancer cells. **a** Immunoblots of TSG101, flotillin-1, HSP70, α-actinin-4, HSP90B1 (also known as GP96, endoplasmin) and calnexin in cellular extracts of ES2, HCT116 and 786-0 cells (Cell), and in lysates of EVs of buoyant densities ranging from 1.09 to 1.13 g/mL that were isolated from media conditioned by these cells (sEV). Uncropped immunoblots are shown in Supplementary Fig. 17. **b** EVs of buoyant densities of 1.09 to 1.13 g/mL were evaluated for particle size distribution by nanoparticle tracking analysis. Each plot shows the combined result of 10 replicate measurements.

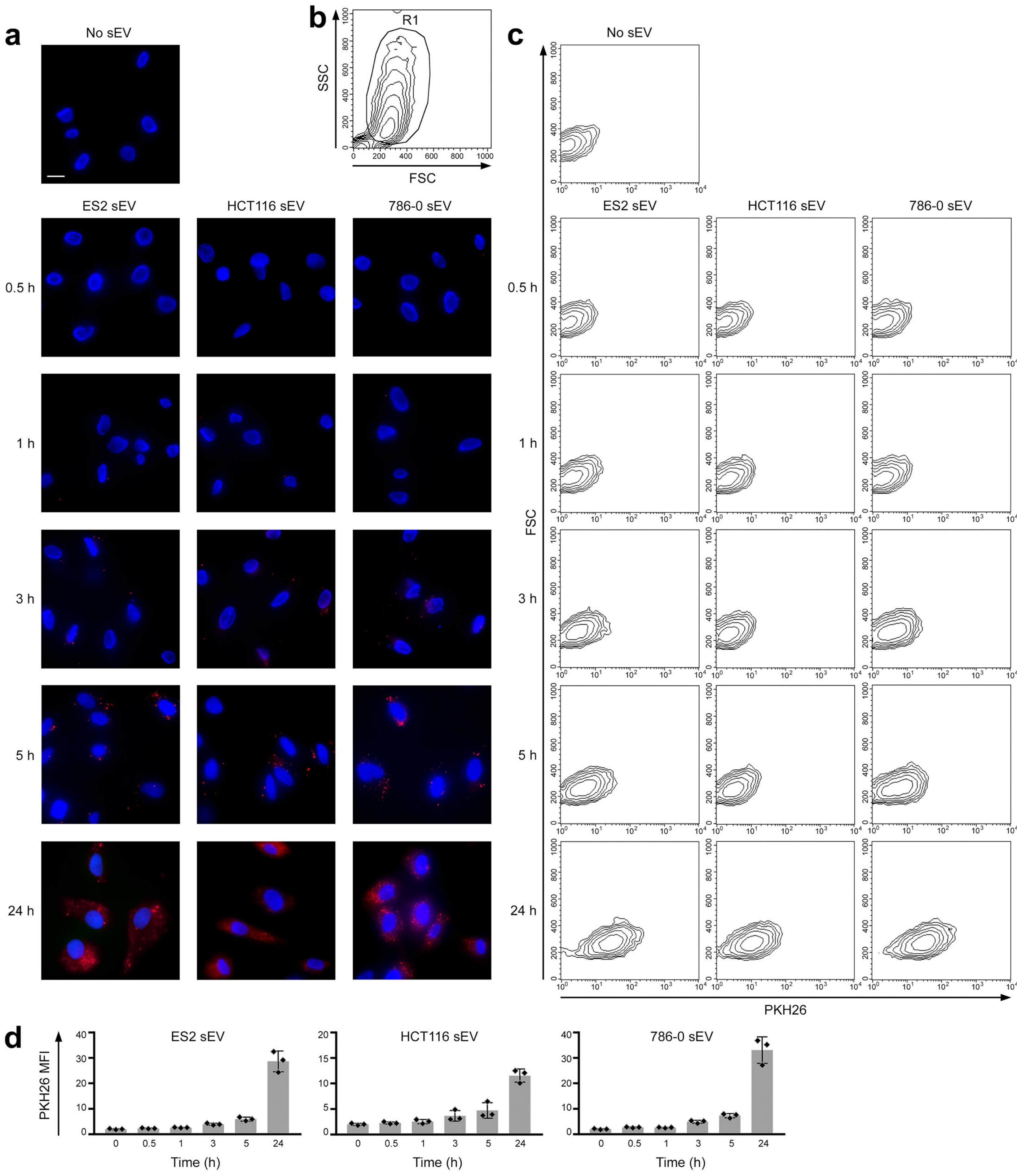

Supplementary Figure 3. Kinetics of sEV uptake. HUVEC were evaluated for uptake of PKH26 dye-labeled sEVs of ES2, HCT116 and 786-0 cells at the indicated times. **a** Representative images of PKH26 fluorescence (red) detected by fluorescence microscopy in HUVEC. Nuclei were visualized by staining with 4',6-diamidino-2-phenyl-indole dihydrochloride (DAPI) (blue). Scale bar=20 μm. **b-d** Evaluation of sEV uptake by flow cytometry. Shown are forward and side scatter analysis indicating the gating used to select the population of live HUVEC within which PKH26 fluorescence was analyzed **(b),** representative contour plots of PKH26 fluorescence detected in HUVEC **(c),** and mean fluorescence intensity (MFI) values of *n*=3 independent experiments (mean ± SD). **(d).** A minimum of 10,000 gated events was analyzed for each sample.

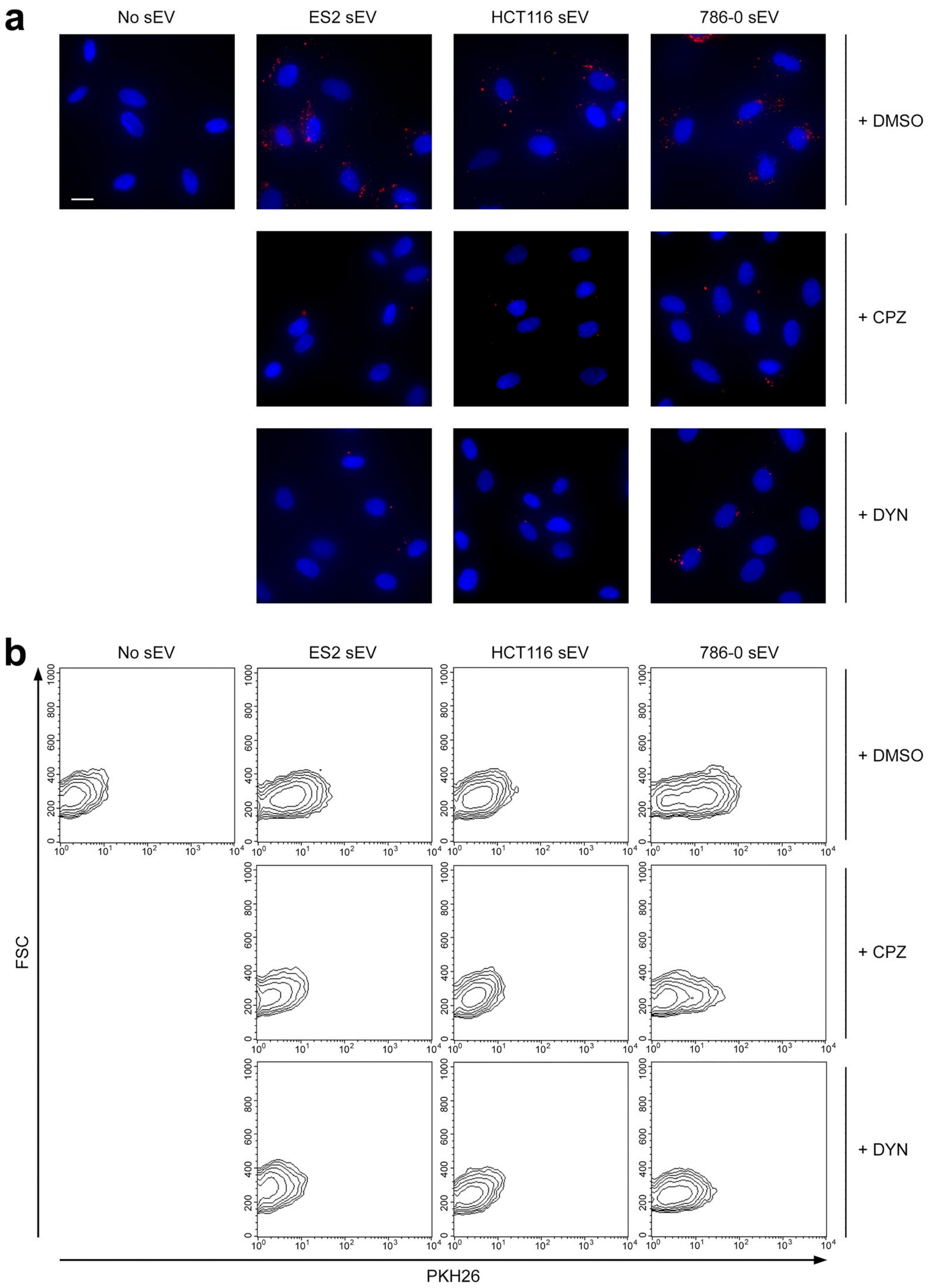

Supplementary Figure 4. Blockade of sEV uptake. HUVEC were pretreated with endocytosis inhibitors (chlorpromazine, CPZ; dynasore, DYN) or with dimethyl sulfoxide (DMSO) solvent, and then incubated with PKH26-labeled sEVs of ES2, HCT116 and 786-0 cells. Uptake of sEVs was evaluated at 5 h after addition of sEVs. **a** Representative images of PKH26 fluorescence (red) detected by fluorescence microscopy in HUVEC. Scale bar=20 μm. **b** Representative contour plots of PKH26 fluorescence detected in HUVEC by flow cytometry.

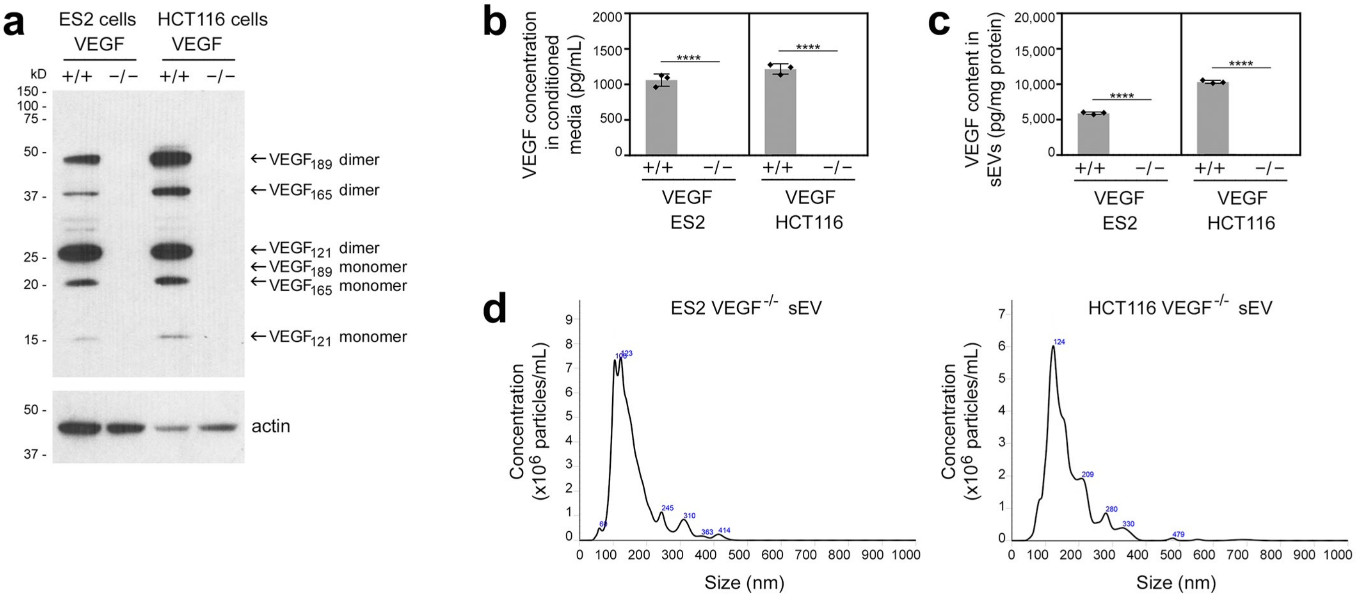

Supplementary Figure 5. Analysis of isogenic VEGF$^{+/+}$ and VEGF$^{-/-}$ cancer cell lines. **a-c** Confirmation of lack of VEGF in ES2 cells in which the *VEGFA* gene was deleted by CRISPR/Cas9 gene editing and in a previously generated HCT116 VEGF$^{-/-}$ cell line[15]. Shown are immunoblots of VEGF in cellular extracts **(a),** levels of VEGF in conditioned media **(b),** and amounts of VEGF in sEVs of parental (VEGF$^{+/+}$) and VEGF$^{-/-}$ cell lines **(c).** Uncropped immunoblots are shown in Supplementary Fig. 17. Mean ± SD of n=3 independent experiments are shown in **b** and **c**. ****P < 0.0001, by two-sided unpaired t-test. **d** Evaluation of particle size distribution of sEVs of VEGF$^{-/-}$ cancer cells by nanoparticle tracking analysis. Each plot shows the combined result of 10 replicate measurements. Particle size distribution of sEVs of parental lines is shown in Supplementary Fig. 2b.

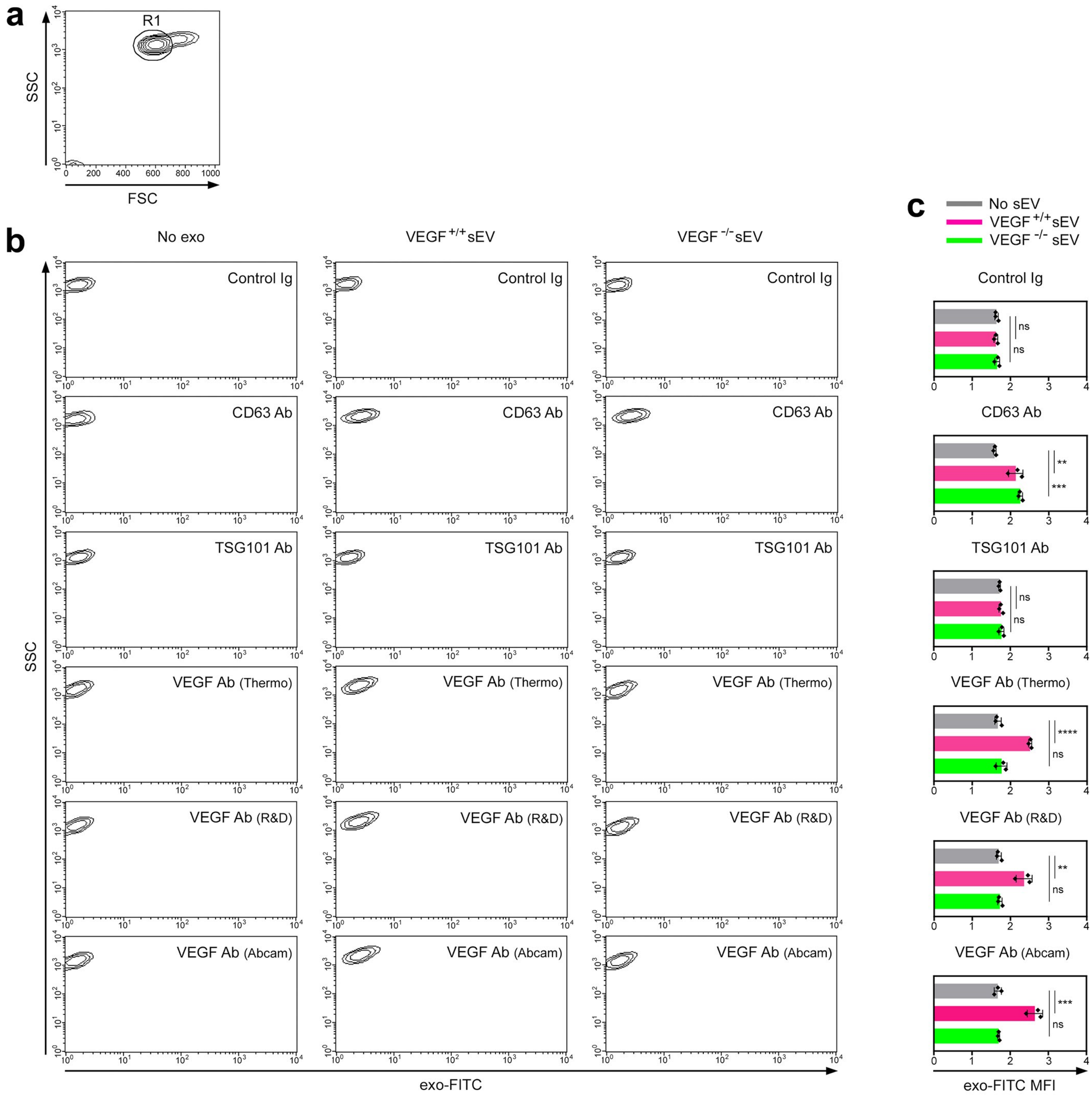

Supplementary Figure 6. Detection of VEGF on the surface of cancer cell-derived sEVs by flow cytometric analysis of binding to Ab-coupled microbeads. Microbeads were coupled to the indicated Ab, incubated with no sEVs or with sEVs of HCT116 $VEGF^{+/+}$ and $VEGF^{-/-}$ cells and then stained with exo-FITC dye to label sEV membrane. Binding of Ab to protein on the surface of sEVs was detected by flow cytometric analysis of exo-FITC fluorescence in the gated population of Ab-coupled microbeads. Detection of VEGF was confirmed by using 3 different commercially available Ab to VEGF. CD63 and TSG101 were assayed as positive and negative controls for sEV surface protein, respectively. **a** Representative example of forward and side scatter analysis indicating the gating used to select the population of singlet Ab-coupled microbeads within which exo-FITC fluorescence was analyzed. A minimum of 10,000 gated events was analyzed for each sample. **b** Representative contour plots of exo-FITC fluorescence. **c** MFI values of *n*=3 independent experiments (mean ± SD). $^{**}P < 0.01$, $^{***}P < 0.001$, $^{****}P < 0.0001$, by one-way ANOVA with Bonferroni's corrections.

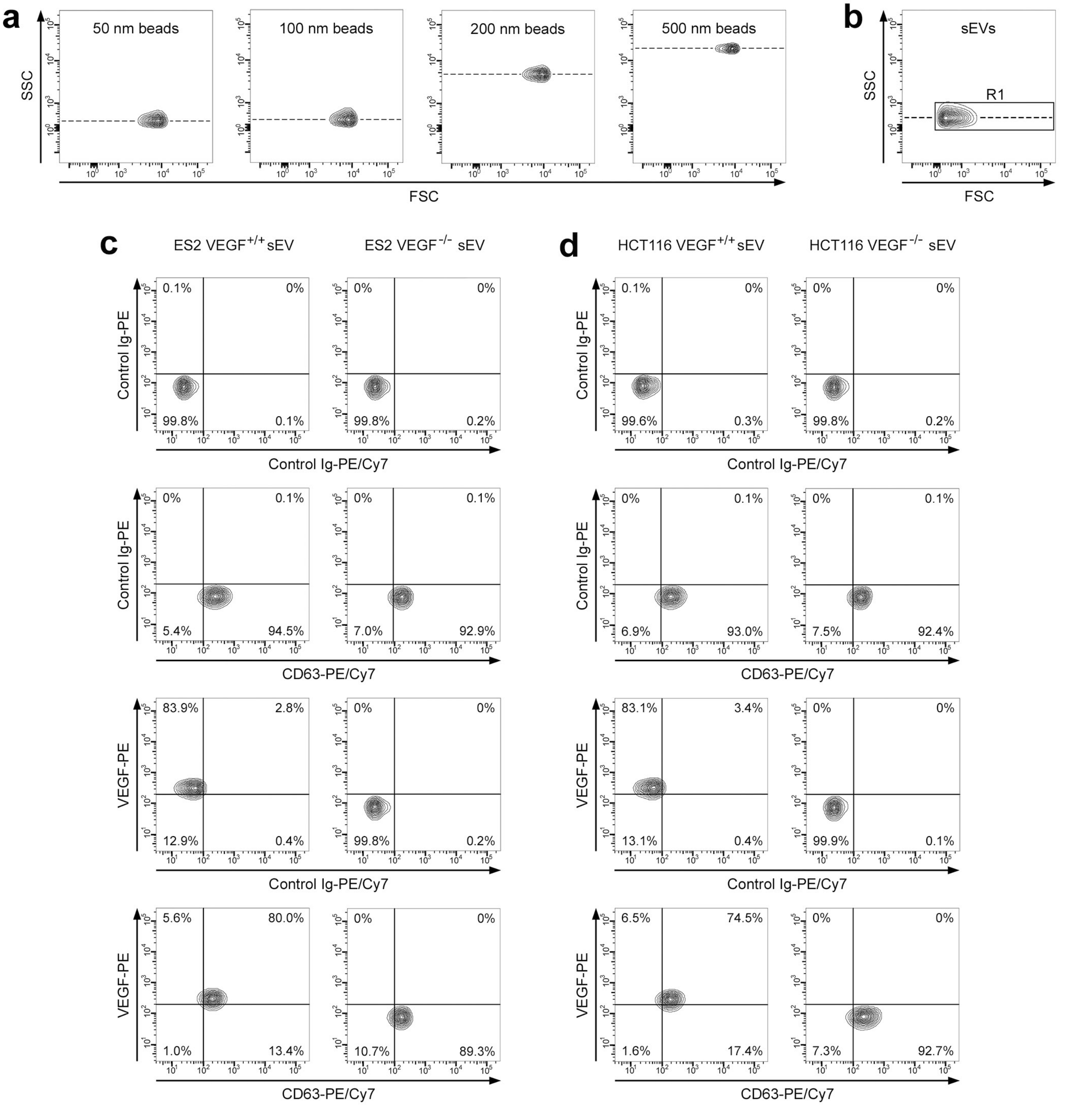

Supplementary Figure 7. Detection of VEGF on the surface of cancer cell-derived sEVs by flow cytometric analysis of direct staining of sEVs. **a** To optimize flow cytometry settings for sEV detection, size marker beads of different diameters (50 nm, 100 nm, 200 nm, 500 nm) were acquired. Shown are forward and side scatter analyses indicating the populations of beads. **b** Isolated sEVs were acquired using the same settings as in **a.** Shown is a representative example of forward and side scatter analysis indicating the gating used to select the population of sEVs. **c-d** sEVs were directly stained with PE-conjugated VEGF Ab or isotype control in combination with PE/Cy7-conjugated CD63 Ab (positive control) or isotype control. PE and PE/Cy7 fluorescence were analyzed within the gated population of sEVs. A minimum of 10,000 gated events was analyzed for each sample. Shown are representative contour plots of staining detected in sEVs of ES2 VEGF$^{+/+}$ and VEGF$^{-/-}$ cells **(c)** and sEVs of HCT116 VEGF$^{+/+}$ and VEGF$^{-/-}$ cells **(d).** Percentages of sEVs in each quadrant are indicated.

Shown in Fig. 3a

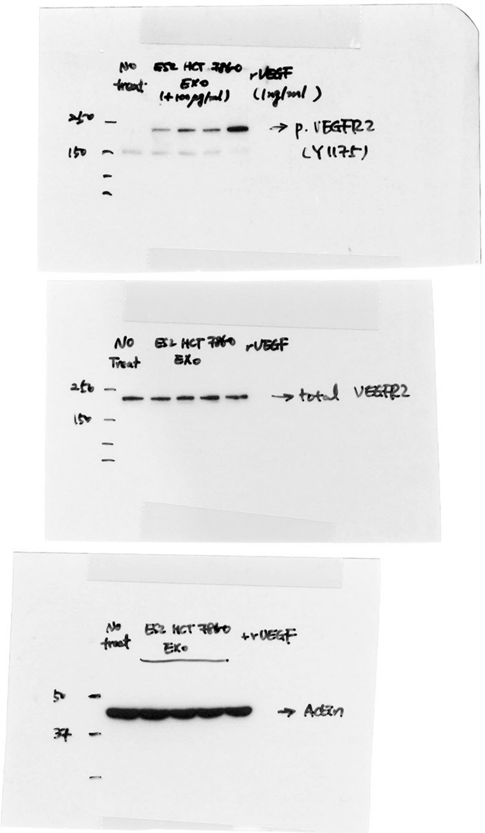

Shown in Fig. 3g

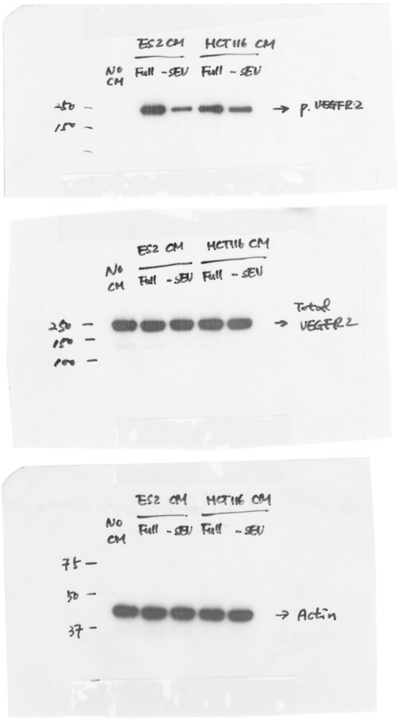

Shown in Fig. 4a

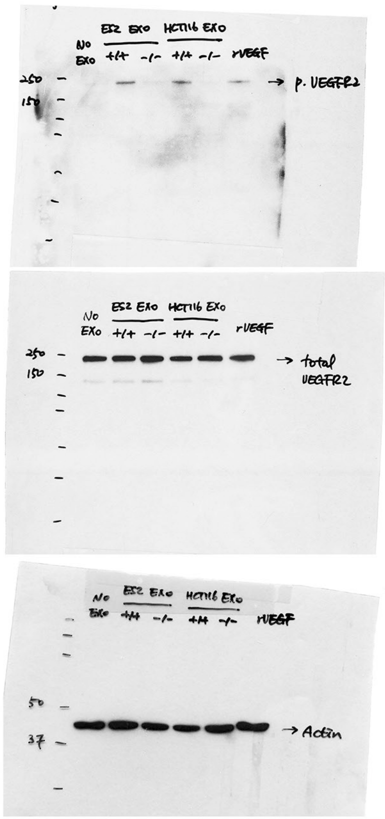

Shown in Fig. 7f

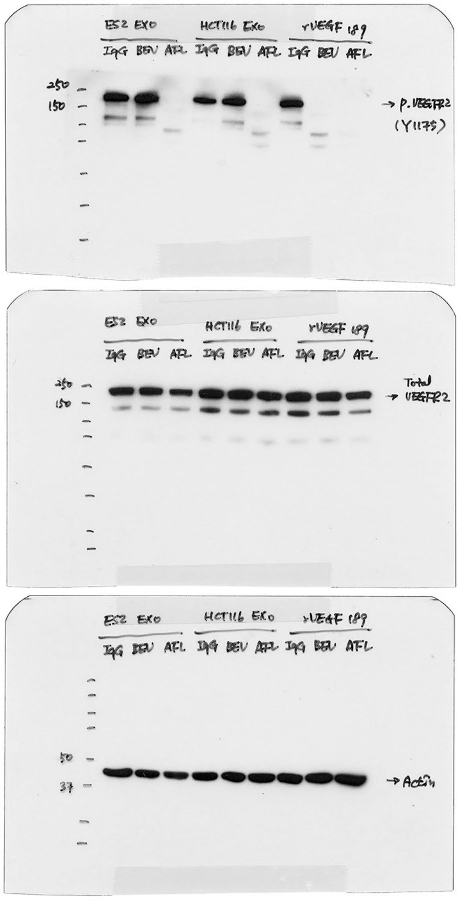

Supplementary Figure 8. Uncropped immunoblots of p-VEGFR2, VEGFR2 and actin shown in Fig. 3a, 3g, 4a and 7f. AFL: VEGFR1/R2-Fc

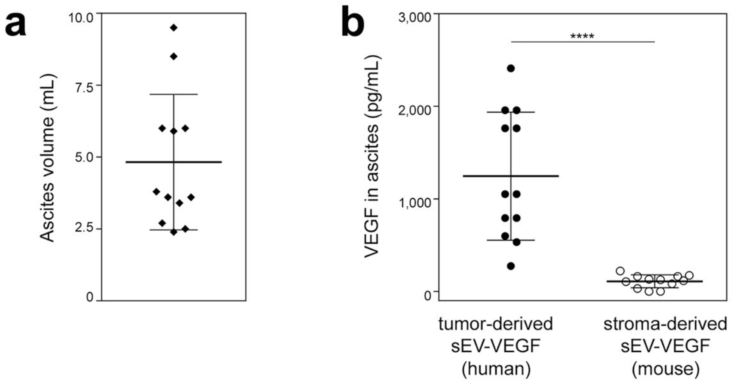

Supplementary Figure 9. Levels of tumor-derived and stroma-derived sEV-VEGF in the parental ES2 xenograft model. Ascites was collected from female nude mice (*n*=12) at 3 weeks following i.p. injection of parental (i.e. $VEGF^{+/+}$) ES2 cells. **a** Total volume of ascites in each mouse. **b** Concentrations of tumor-derived sEV-VEGF and stroma-derived sEV-VEGF in ascites of each mouse were determined by isolating sEVs from ascites and then assaying VEGF in sEVs by human- and mouse- specific VEGF ELISA, respectively. ****$P < 0.0001$, by two-sided paired *t*-test.

Shown in Fig. 5a

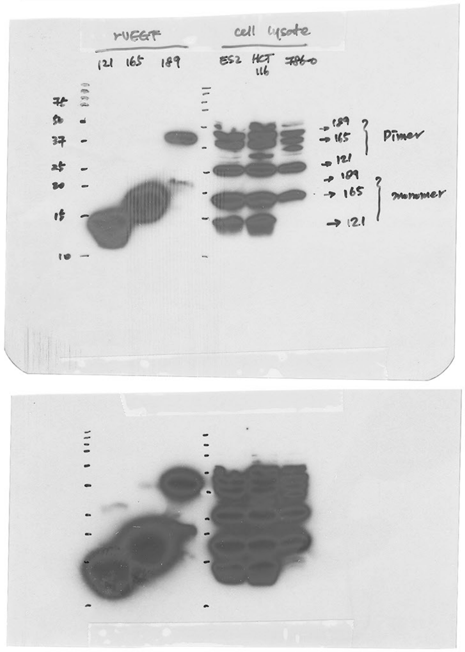

overexposure

Shown in Fig. 5b

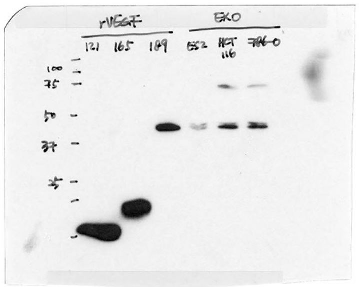

Shown in Fig. 5c

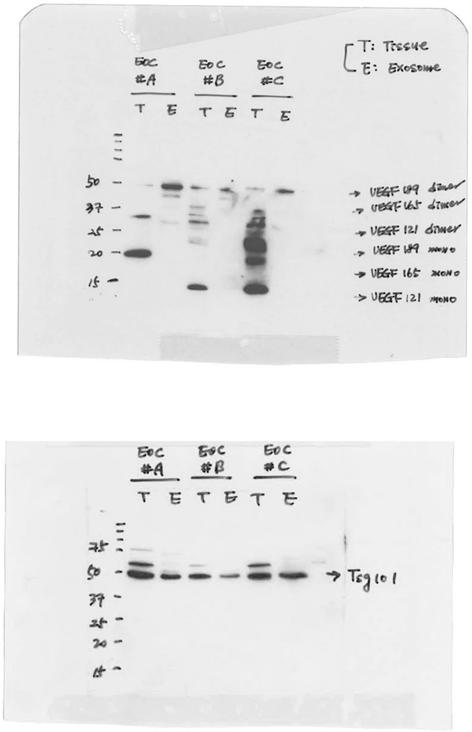

Shown in Fig. 5d

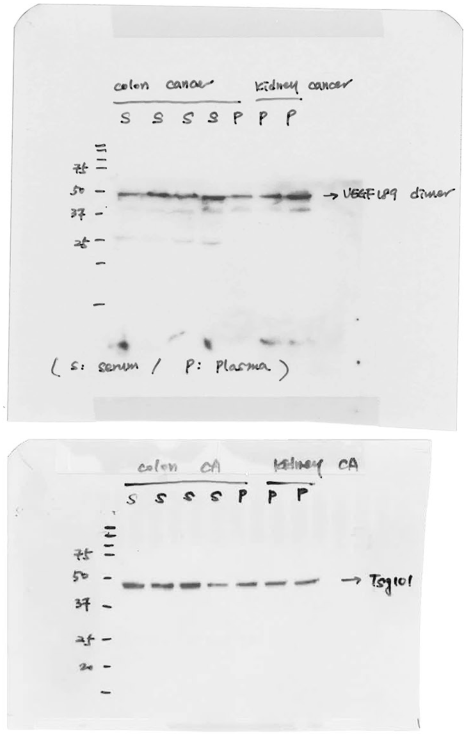

Supplementary Figure 10. Uncropped immunoblots of VEGF shown in Fig. 5a and 5b, and of VEGF and TSG101 shown in Fig. 5c and 5d.

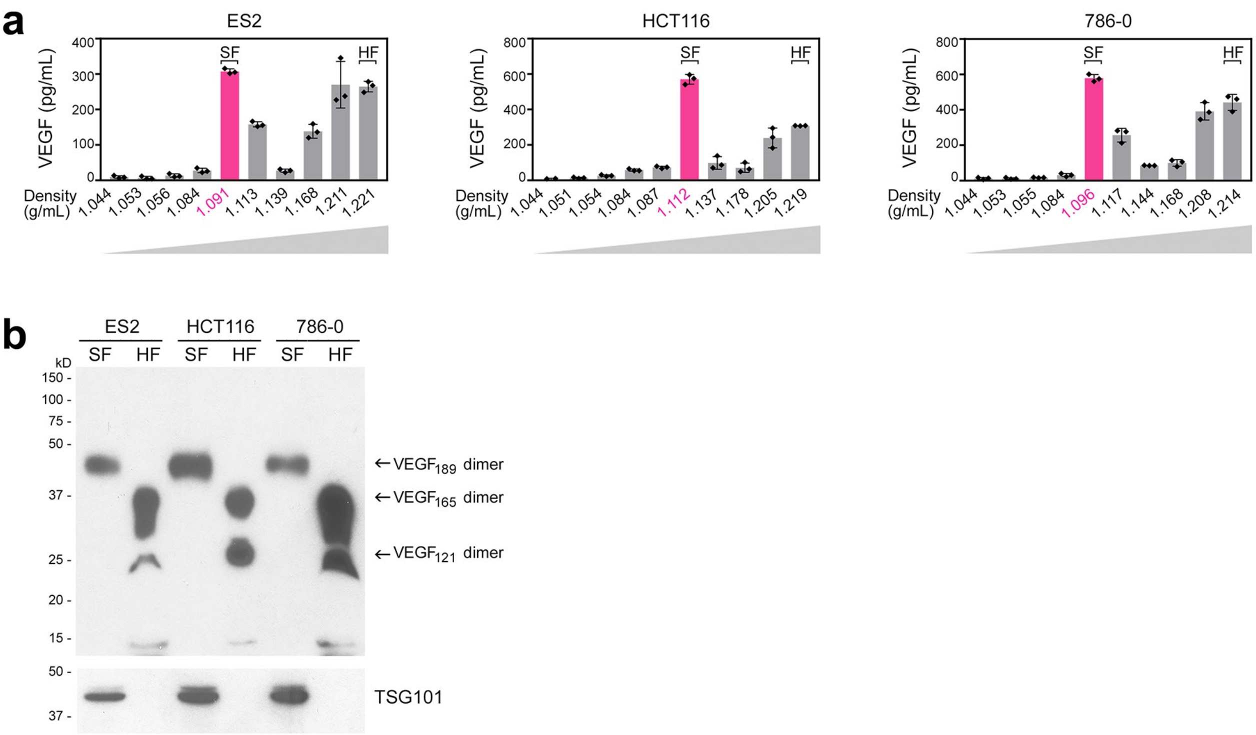

Supplementary Figure 11. Analysis of VEGF in fractions isolated by density gradient ultracentrifugation. **a** Fractions of the indicated buoyant densities (1.0 mL in volume) were isolated by density gradient ultracentrifugation from media conditioned by ES2, HCT116 and 786-0 cells. A 100 μL aliquot of each fraction was evaluated for VEGF content by ELISA. Shown are mean ± SD of *n*=3 independent assays. **b** Immunoblot of VEGF in fractions within the buoyant density range of sEVs (SF) and in fractions of the highest buoyant density (HF). Specific fractions are indicated in **a**. Equivalent volumes of each fraction (40 μL) were assayed. TSG101 was assayed as a control. Uncropped immunoblots are shown in Supplementary Fig. 17.

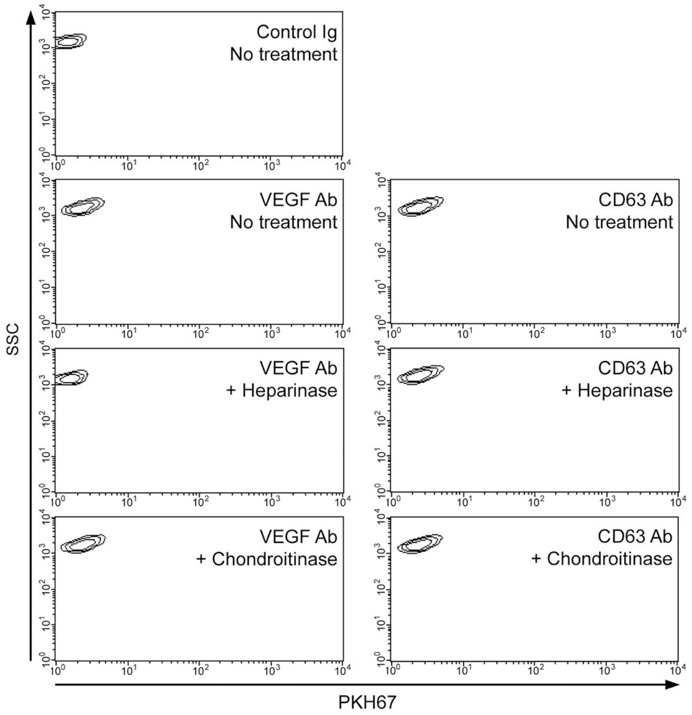

Supplementary Figure 12. Effect of enzymatic digestion on association of VEGF with the surface of sEVs. PKH67-labeled sEVs of parental ES2 cells were pretreated with heparinase, chondroitinase or no enzyme, and then incubated with VEGF Ab coupled to microbeads. VEGF on the surface of sEVs was detected by flow cytometric analysis of PKH67 fluorescence in the gated population of Ab-coupled microbeads. Gating strategy is shown in Supplementary Fig. 6a. As a negative control for enzymatic digestion, the same approach was used to detect the transmembrane protein CD63. Shown are representative contour plots of PKH67 fluorescence.

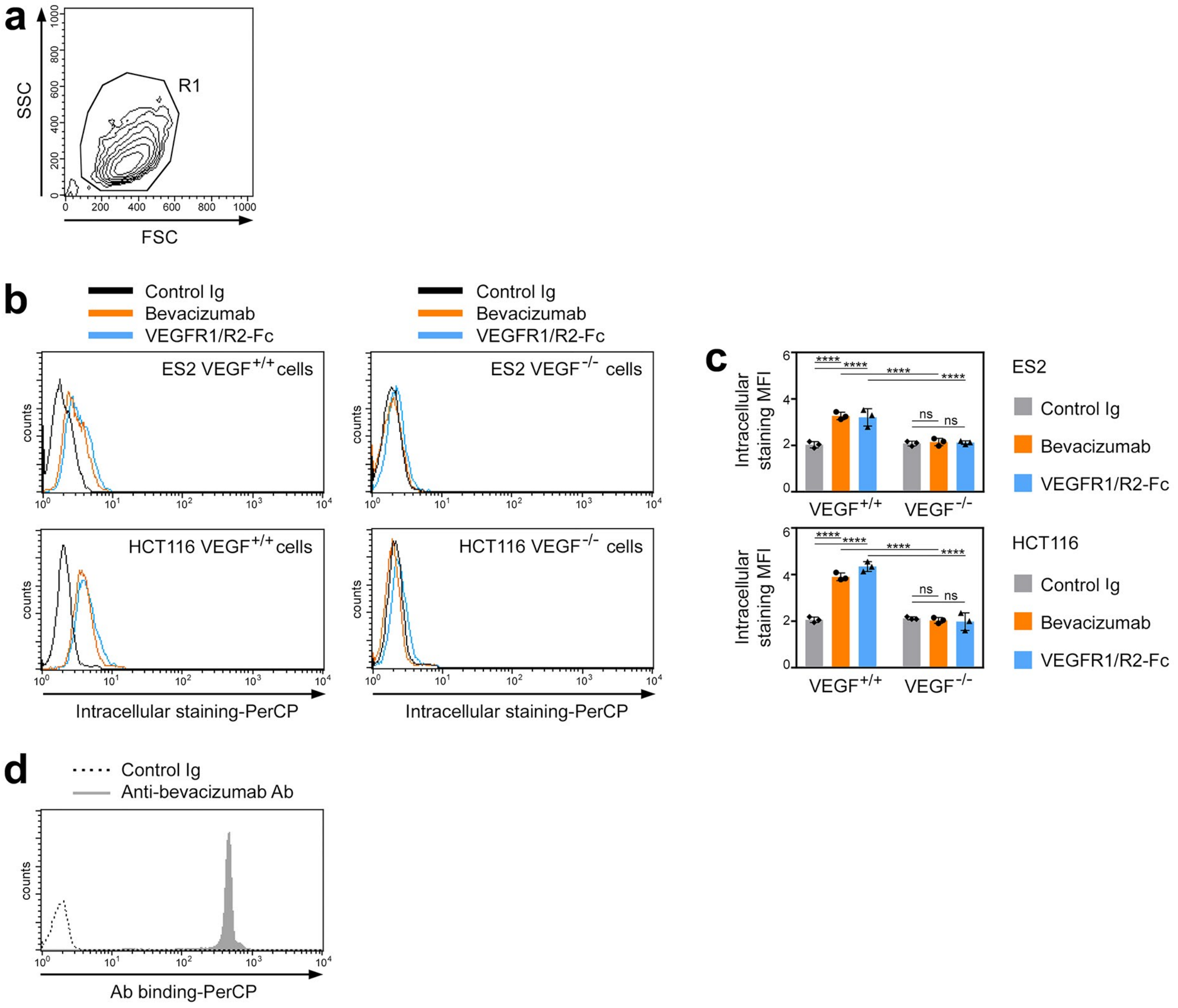

Supplementary Figure 13. Control assays to detect bevacizumab binding. **a-c** To confirm that binding of bevacizumab to VEGF can be detected by flow cytometry, isogenic VEGF$^{+/+}$ and VEGF$^{-/-}$ cancer cells were treated with brefeldin A, permeabilized and then incubated with control Ig, bevacizumab or VEGFR1/R2-Fc. Binding to intracellular VEGF was detected by staining with PerCP-conjugated anti-human Ig. Shown are a representative example of forward and side scatter analysis indicating the gating used to select the population of permeabilized cancer cells within which intracellular staining was analyzed **(a),** representative histogram plots of intracellular staining **(b),** and MFI values of n=3 independent experiments (mean ± SD). **(c).** ****P < 0.0001, by two-way ANOVA with Bonferroni's corrections. A minimum of 10,000 gated events was analyzed for each sample. **d** To confirm that bevacizumab was coupled to microbeads that were used for incubation with sEVs, microbeads were stained with anti-bevacizumab Ab and staining evaluated by flow cytometry (solid histogram). Dotted histogram indicates staining of beads with isotype control.

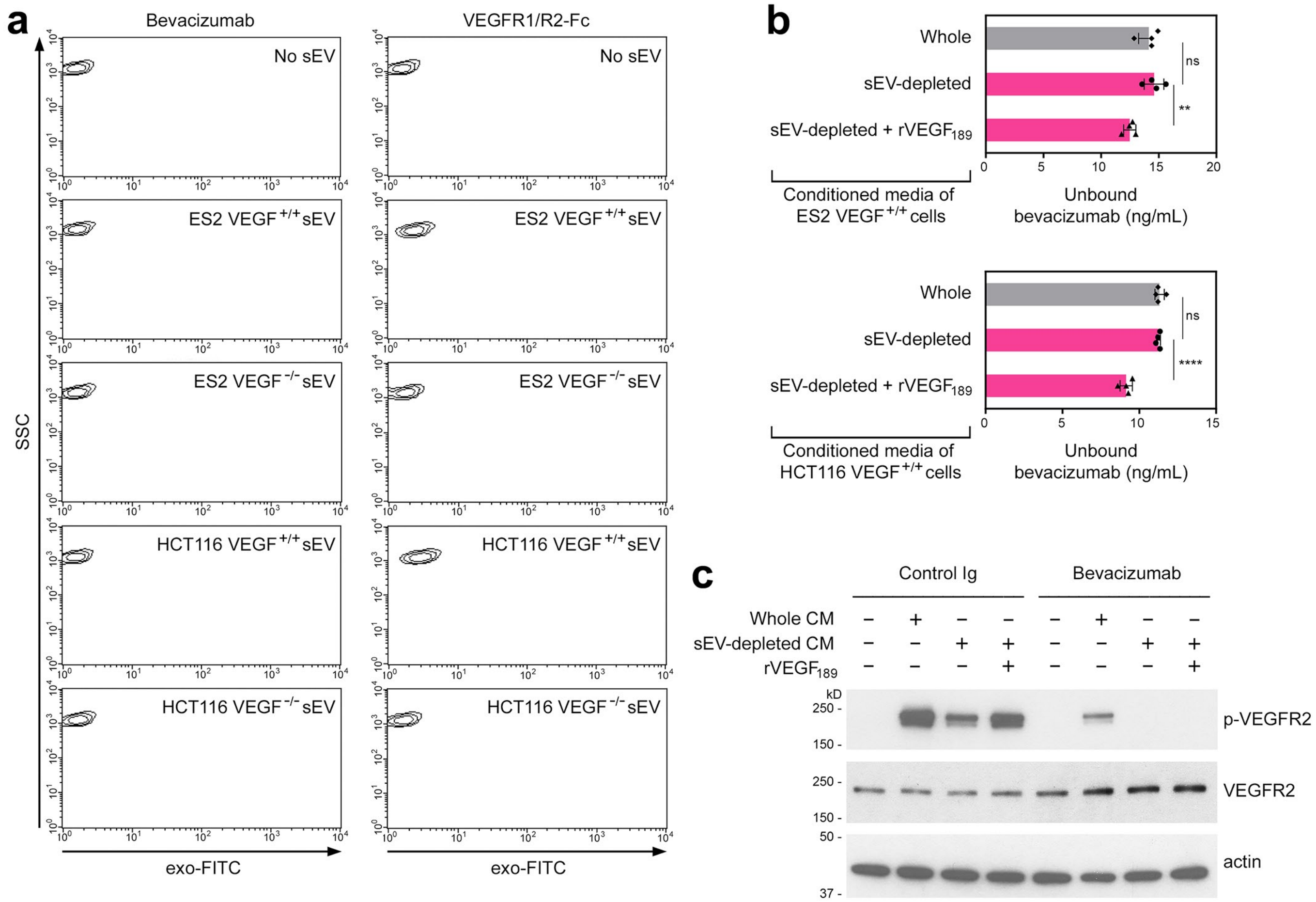

Supplementary Figure 14. Analysis of ability of bevacizumab to neutralize sEV-VEGF *in vitro*. **a** To evaluate the ability of bevacizumab to bind sEV-VEGF, microbeads were coupled to bevacizumab, incubated with sEVs of VEGF$^{+/+}$ cells or sEVs of VEGF$^{-/-}$ cells (negative control) and then stained with exo-FITC dye to label sEV membrane. The same procedure was performed using microbeads coupled to VEGFR1/R2-Fc (positive control). Binding of bevacizumab and VEGFR1/R2-Fc to sEV-VEGF was assayed by flow cytometric analysis of exo-FITC fluorescence in the gated population of microbeads. Gating strategy is shown in Supplementary Fig. 6a. A minimum of 10,000 gated events was analyzed for each sample. Shown are representative contour plots of exo-FITC fluorescence. **b-c** To evaluate the ability of bevacizumab to neutralize sEV-VEGF under conditions where soluble VEGF and sEVs carrying VEGF are co-secreted, conditioned media was collected from parental ES2 and HCT116 cells and either depleted of sEVs or left non-depleted (i.e. whole). As a control, rVEGF$_{189}$ was added to sEV-depleted media at a concentration equivalent to the concentration of sEV-VEGF in whole media (i.e. 400 pg/mL, refer Fig. 3f). In **b,** whole and sEV-depleted media were incubated with bevacizumab (20 ng/mL) and thereafter assayed for levels of unbound bevacizumab. Shown are mean ± SD of n=4 independent experiments. $^{**}P < 0.01$, $^{****}P < 0.0001$, by one-way ANOVA with Bonferroni's corrections. In **c,** HUVEC were stimulated with whole and sEV-depleted conditioned media (CM) of parental ES2 cells with the addition of bevacizumab or control Ig, and then assayed for VEGFR2 phosphorylation by immunoblot. Uncropped immunoblots are shown in Supplementary Fig. 17.

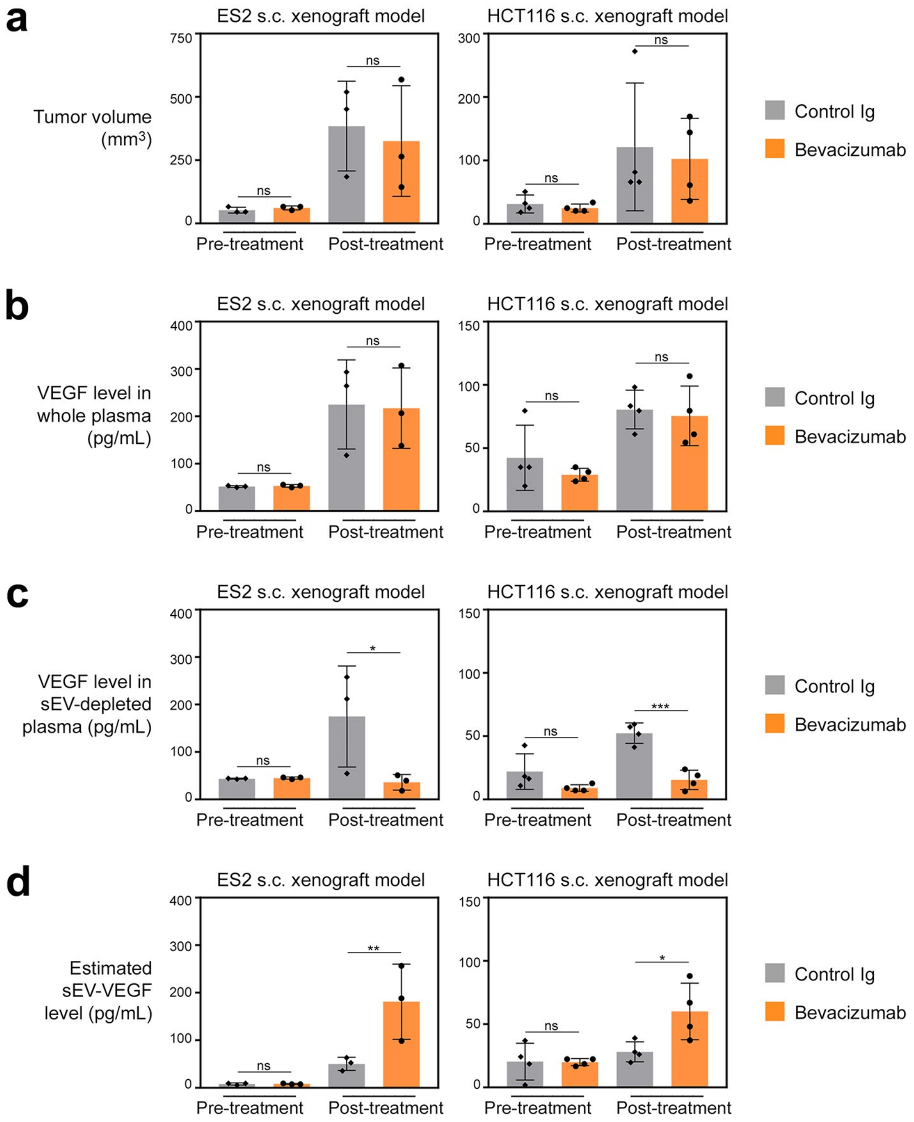

Supplementary Figure 15. Analysis of ability of bevacizumab to neutralize naturally secreted sEV-VEGF *in vivo*. Nude mice were inoculated s.c. with parental ES2 or HCT116 cells. At 7 days thereafter when tumors were palpable, peripheral blood samples were collected (i.e. pre-treatment). Mice were then randomized into groups (n=3 per group for ES2; n=4 per group for HCT116) and administered either normal human IgG (control) or bevacizumab i.p. 3 times for one week. Thereafter, peripheral blood samples were again collected (i.e. post-treatment). Pre- and post-treatment plasma samples were depleted of sEVs or left non-depleted (i.e. whole) and assayed for human VEGF by ELISA. **a** Volumes of s.c. tumors at pre- and post- treatment time-points. **b** VEGF levels in whole plasma. **c** VEGF levels in sEV-depleted plasma (representing non-sEV-VEGF). **d** Levels of sEV-VEGF estimated from the difference between VEGF levels in whole and sEV-depleted plasma. *$P < 0.05$, **$P < 0.01$, ***$P < 0.001$, by one-way ANOVA with Bonferroni's corrections.

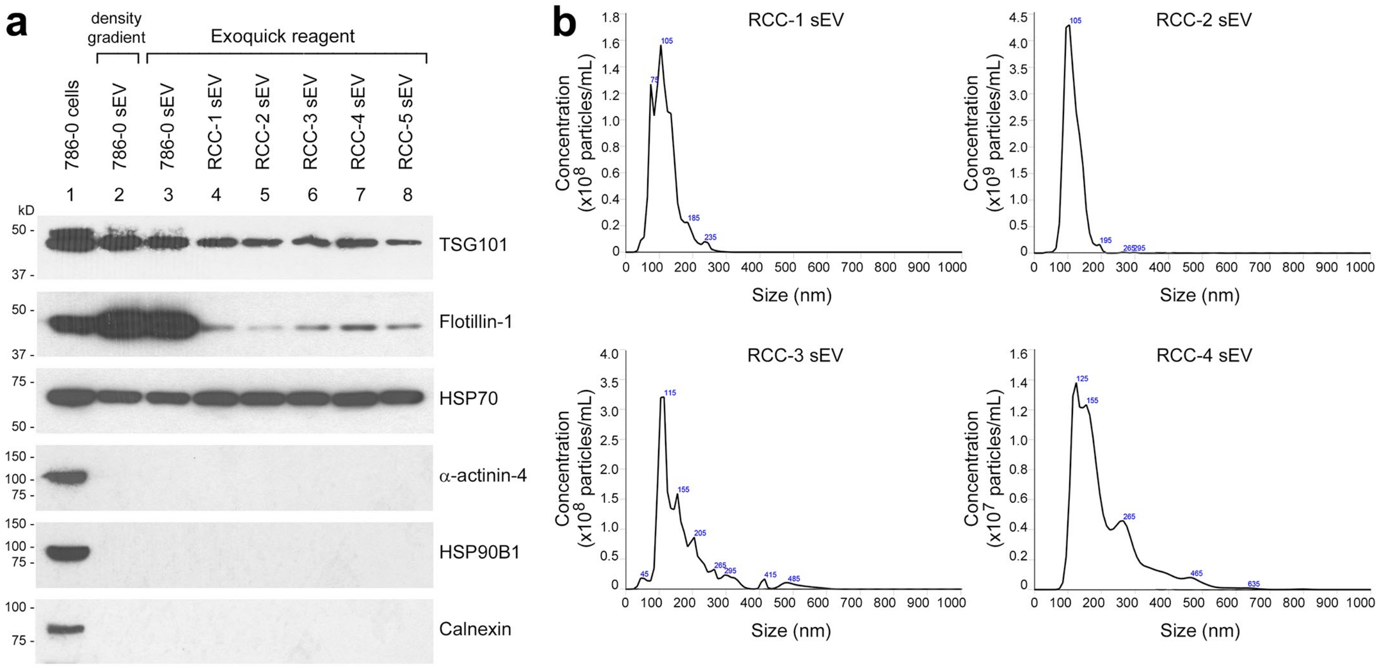

Supplementary Figure 16. Analysis of markers and particle size distribution of plasma sEVs of bevacizumab-treated patients. sEVs were isolated from plasma samples from a Phase II trial of patients with newly diagnosed metastatic renal cell carcinoma who had been treated presurgically with bevacizumab (study NCT00113217)[29]. Because volumes of these plasma samples were too small for sEVs to be isolated by density gradient ultra-centrifugation, sEVs were isolated from these samples by using ExoQuick reagent. **a** Immunoblots of TSG101, flotillin-1, HSP70, α-actinin-4, HSP90B1 and calnexin in sEVs isolated from plasma samples of 5 patients (*lanes 4 to 8*). Included as controls are sEVs that were isolated from 786-0 renal cancer cells by using ExoQuick reagent (*lane 3*) and by density gradient ultracentrifugation (*lane 2*). Cellular extract of 786-0 cells was included as a positive control for non-sEV markers (*lane 1*). Uncropped immunoblots are shown in Supplementary Fig. 17. **b** Evaluation of particle size distribution of sEVs isolated from plasma samples of 4 patients by nanoparticle tracking analysis. Each plot shows the combined result of 10 replicate measurements.

Shown in Supplementary Fig. 2a

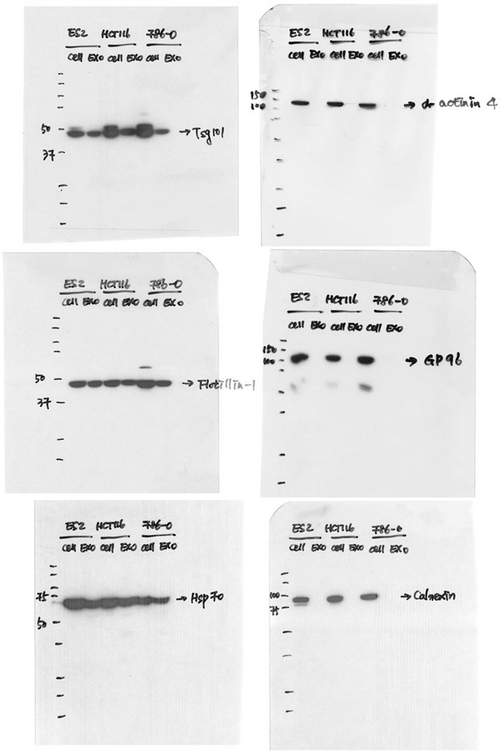

Shown in Supplementary Fig. 5a

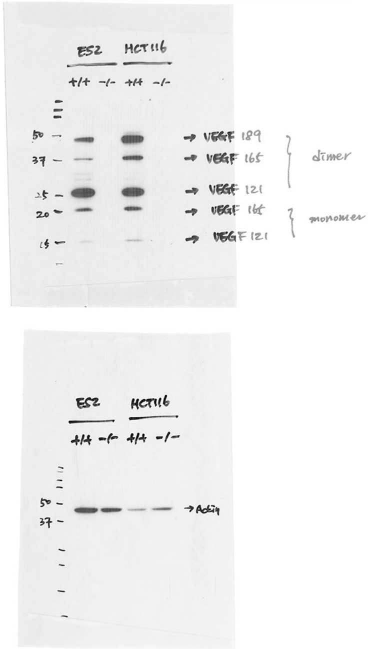

Shown in Supplementary Fig. 11b

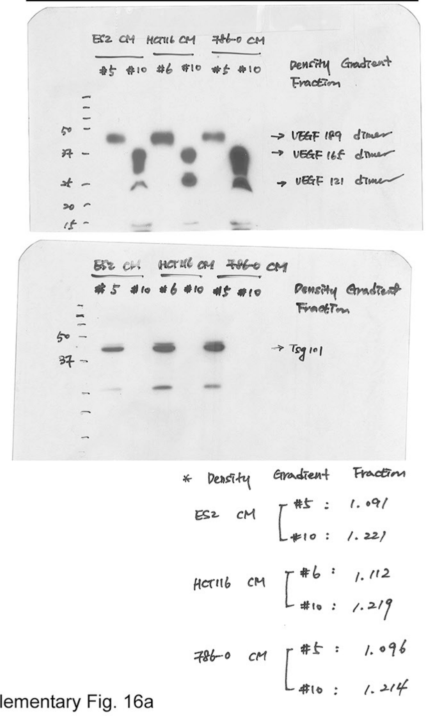

Shown in Supplementary Fig. 14c

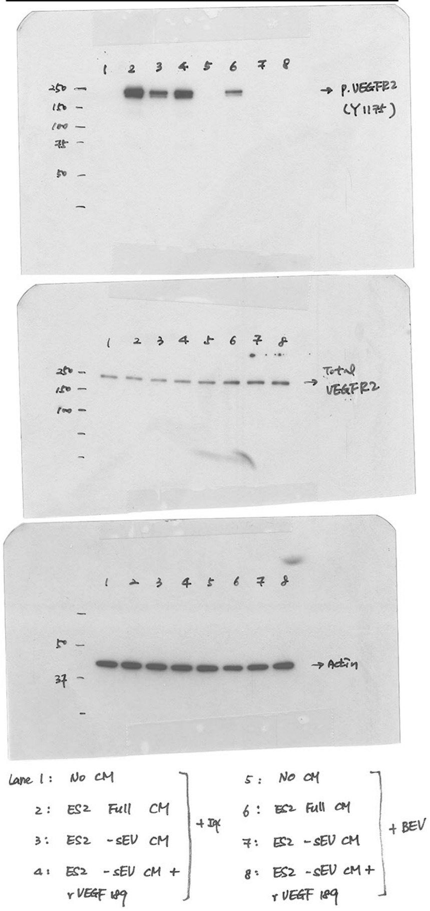

Shown in Supplementary Fig. 16a

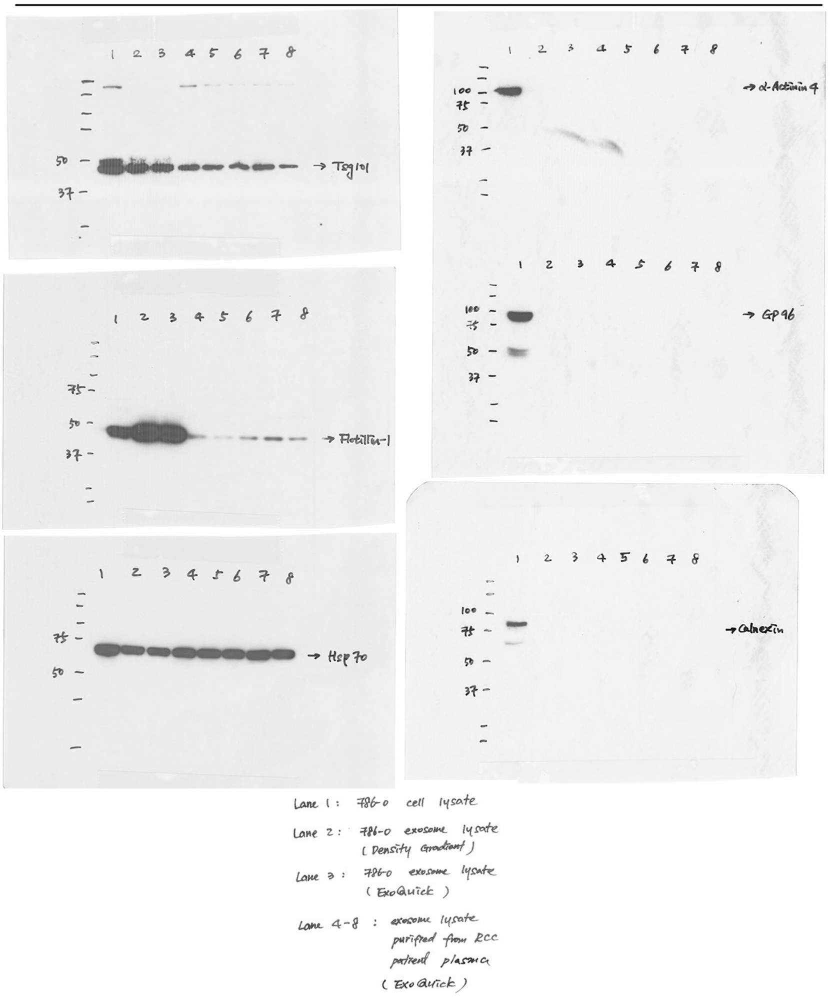

Supplementary Figure 17. Uncropped immunoblots of TSG101, flotillin-1, HSP70, α-actinin-4, HSP90B1 (GP96) and calnexin shown in Supplementary Fig. 2a, VEGF and actin shown in Supplementary Fig. 5a, VEGF and TSG101 shown in Supplementary Fig. 11b, p-VEGFR2, VEGFR2 and actin shown in Supplementary Fig. 14c, and TSG101, flotillin-1, HSP70, α-actinin-4, HSP90B1 (GP96) and calnexin shown in Supplementary Fig. 16a.
