# Supplementary material for: Cancer-derived small extracellular vesicles promote angiogenesis by heparin-bound, bevacizumab-insensitive VEGF, independent of vesicle uptake
Source: Commun Biol. 2019 Oct 18;2:386. doi: 10.1038/s42003-019-0609-x (PMC6802217; doi:10.1038/s42003-019-0609-x)
Supplement: Supplementary file 2 — Description of additional supplementary files [file 42003_2019_609_MOESM2_ESM.docx]

**Description of additional supplementary files**

**Supplementary Data 1.** Source data used for graphs shown in Figure 1.

**Supplementary Data 2.** Source data used for graphs shown in Figure 2.

**Supplementary Data 3.** Source data used for graphs shown in Figure 3 and for Table 1.

**Supplementary Data 4.** Source data used for graphs shown in Figure 4.

**Supplementary Data 5.** Source data used for graphs shown in Figure 6.

**Supplementary Data 6.** Source data used for graphs shown in Figure 7.

**Supplementary Data 7.** Source data used for graphs shown in Figure 8.
