# Supplementary material for: Cancer-derived small extracellular vesicles promote angiogenesis by heparin-bound, bevacizumab-insensitive VEGF, independent of vesicle uptake
Source: Commun Biol. 2019 Oct 18;2:386. doi: 10.1038/s42003-019-0609-x (PMC6802217; doi:10.1038/s42003-019-0609-x)
Supplement: Supplementary file 10 — Reporting Summary [file 42003_2019_609_MOESM10_ESM.pdf]

nature research

Corresponding author(s): Honami Naora

Last updated by author(s): Sep 6, 2019

Reporting Summary

Nature Research wishes to improve the reproducibility of the work that we publish. This form provides structure for consistency and transparency in reporting. For further information on Nature Research policies, see Authors & Referees and the Editorial Policy Checklist .

Statistics

For all statistical analyses, confirm that the following items are present in the figure legend, table legend, main text, or Methods section.

n/a	Confirmed	
☐	☒	The exact sample size (*n*) for each experimental group/condition, given as a discrete number and unit of measurement
☐	☒	A statement on whether measurements were taken from distinct samples or whether the same sample was measured repeatedly
☐	☒	The statistical test(s) used AND whether they are one- or two-sided<br>*Only common tests should be described solely by name; describe more complex techniques in the Methods section.*
☐	☒	A description of all covariates tested
☐	☒	A description of any assumptions or corrections, such as tests of normality and adjustment for multiple comparisons
☐	☒	A full description of the statistical parameters including central tendency (e.g. means) or other basic estimates (e.g. regression coefficient) AND variation (e.g. standard deviation) or associated estimates of uncertainty (e.g. confidence intervals)
☐	☒	For null hypothesis testing, the test statistic (e.g. *F*, *t*, *r*) with confidence intervals, effect sizes, degrees of freedom and *P* value noted<br>*Give P values as exact values whenever suitable.*
☒	☐	For Bayesian analysis, information on the choice of priors and Markov chain Monte Carlo settings
☒	☐	For hierarchical and complex designs, identification of the appropriate level for tests and full reporting of outcomes
☐	☒	Estimates of effect sizes (e.g. Cohen's *d*, Pearson's *r*), indicating how they were calculated

Our web collection on statistics for biologists contains articles on many of the points above.

Software and code

Policy information about availability of computer code

Data collection	ND-1000 software (for spectrophotometric readings, Thermo Scientific), Gen 5 software (for microplate readings, BioTek), Picture Frame software (for capturing mouse images, Optronics), NIS Element BR 4 software (for all other microscopic imaging, Nikon), BD CellQuest Pro and FACS Diva software (for flow cytometry, BD Biosciences).
Data analysis	Statistical analysis of data was performed by using STATISTICA13.1 software (StatSoft Inc.) and GraphPad Prism 8 software (GraphPad Software Inc.). For analyzing tube formation, the number of tubes in each field was quantified by using NIH ImageJ software with Angiogenesis Analyzer plugin. Analysis of flow cytometry data was performed by using BD CellQuest Pro (BD Biosciences) and FlowJo v10.6.0 (FlowJo LLC) software. Analysis of areas of fluorescence in mice was performed by using Image Pro Plus software (Media Cybernetics).

For manuscripts utilizing custom algorithms or software that are central to the research but not yet described in published literature, software must be made available to editors/reviewers. We strongly encourage code deposition in a community repository (e.g. GitHub). See the Nature Research guidelines for submitting code & software for further information.

Data

Policy information about availability of data

All manuscripts must include a data availability statement. This statement should provide the following information, where applicable:

- Accession codes, unique identifiers, or web links for publicly available datasets
- A list of figures that have associated raw data
- A description of any restrictions on data availability

The authors declare that all data supporting the findings of this study are available within the manuscript and its supplementary information files.

Field-specific reporting

Please select the one below that is the best fit for your research. If you are not sure, read the appropriate sections before making your selection.

- [x] Life sciences
- [ ] Behavioural & social sciences
- [ ] Ecological, evolutionary & environmental sciences

For a reference copy of the document with all sections, see nature.com/documents/nr-reporting-summary-flat.pdf

Life sciences study design

All studies must disclose on these points even when the disclosure is negative.

Sample size	1) Animal studies: Based on the variance of i.p. xenograft growth that was observed in control mice in preliminary studies, power calculations indicated the use of six mice per group to detect a difference of >39% in i.p. tumor burden, numbers of intratumoral endothelial cells and ascites volume between groups in a two-sided test at a significance of $P < 0.05$ and with 80% probability.<br><br>2) Human clinical specimen studies: Sample size was limited by availability of archived specimens. To analyze VEGF isoforms in sEVs, a minimum volume of 1.0 mL of biological fluid (ascites, serum or plasma) was required. Samples of the required volume were only available for 3, 5 and 2 cases of ovarian, colorectal and renal cell carcinoma, respectively. Of the 27 patients with newly diagnosed metastatic renal cell carcinoma who were treated presurgically with single-agent bevacizumab and thereafter restaged (study NCT00113217), pretreatment plasma samples were available for 17 evaluable patients with Stage IV disease.
Data exclusions	1) Animal studies: Mice that did not develop i.p. tumors at 21 days or s.c. tumors at 14 days following inoculation of tumor cells were excluded from analysis.<br><br>2) Human clinical specimen studies: All data were included.
Replication	All efforts to replicate the experiments were successful.
Randomization	1) Animal studies: Mice were inoculated with tumor cells and at 7 days thereafter were allocated randomly into treatment groups.<br><br>2) Human clinical specimen studies: Randomization was not applicable.
Blinding	1) Animal studies: The investigators were not blinded to the allocation of mouse groups during experiments because the groups needed to be administered with different agents.<br><br>2) Human clinical specimen studies: All assays of VEGF levels in clinical specimens of human biological fluids were performed blinded to clinical data.

Reporting for specific materials, systems and methods

We require information from authors about some types of materials, experimental systems and methods used in many studies. Here, indicate whether each material, system or method listed is relevant to your study. If you are not sure if a list item applies to your research, read the appropriate section before selecting a response.

Materials & experimental systems

n/a	Involved in the study	
[ ]	[x]	Antibodies
[ ]	[x]	Eukaryotic cell lines
[x]	[ ]	Palaeontology
[ ]	[x]	Animals and other organisms
[ ]	[x]	Human research participants
[x]	[ ]	Clinical data

Methods

n/a	Involved in the study	
[x]	[ ]	ChIP-seq
[ ]	[x]	Flow cytometry
[x]	[ ]	MRI-based neuroimaging

Antibodies

Antibodies used	1) For flow cytometry: anti-VEGF (R&D Systems, #mab2931, 1 microgram/test; Thermo Fisher Scientific, #P802, 1 microgram/test; Abcam, #ab52917, 1 microgram/test); PE-anti-VEGF (Abcam, #ab209439, 1:100 dilution); bevacizumab (Genentech, 1 microgram/test); VEGFR1/R2-Fc (Aflibercept, Regeneron Pharmaceuticals, 1 microgram/test); anti-bevacizumab (Abnova, #mab11128, 1:100 dilution); anti-TSG101 (Abcam, #ab125011, 1 microgram/test); anti-CD63 (BD Biosciences, #556019, 1 microgram/test); PE/Cy7-anti-CD63 (BioLegend, #353010, 1:100 dilution); PerCP-anti-human IgG Fc (BioLegend, #409312, 1:100 dilution); PE/Cy7-anti-mouse IgG1 (BioLegend, #400126, 1:100 dilution); PE-anti-rabbit IgG (Santa Cruz Biotechnology, #sc-3739, 1:100 dilution); PerCP-anti-mouse IgG1 (BD, #340272, 1:100 dilution); biotinylated secondary antibodies (Abcam, #ab97263, ab97198, #ab97223, 1 microgram/test).

2) For immunoblot: anti-VEGFR2 (Cell Signaling Technology, #9698, 1:1000 dilution); anti-phospho-VEGFR2 (Tyr1175) (Cell Signaling Technology, #3770, 1:1000 dilution); anti-VEGF (Abcam, #ab183100, 1:1000 dilution); anti-flotillin-1 (BD Biosciences, #610820, 1:1000 dilution); anti-TSG101 (Abcam, #ab125011, 1:1000 dilution); anti-HSP70 (BD Biosciences, #610607, 1:2000 dilution); anti-HSP90B1 (Enzo Life Sciences, ADI-SPA-850, 1:1000 dilution); anti-alpha-actinin 4 (Abcam, #ab108198, 1:1000 dilution); anti-calnexin (Cell Signaling, #2679, 1:1000 dilution); anti-actin (Sigma, #A1978, 1:5000 dilution); HRP-conjugated secondary antibodies (Jackson ImmunoResearch, #115-035-166, #111-035-144, 1:5000 dilution)

3) For immunogold labeling: anti-CD63 (System Biosciences, #EXOAB-CD63A-1, 1:5 dilution); anti-VEGF (Thermo Fisher Scientific, #P802, 1:10 dilution); 10nm goat-anti-rabbit IgG gold conjugate (Electron Microscopy Sciences, #25109, 1:20 dilution)

4) For ELISA: bevacizumab (Genentech, 1 microgram/mL); VEGFR1/R2-Fc (Aflibercept, Regeneron Pharmaceuticals, 1 microgram/mL); anti-VEGF for capture (R&D Systems, #mab293, 1 microgram/mL); HRP-conjugated anti-VEGF for detection (R&D Systems, #890219, 100ng/mL); human GRO alpha/IL-8/FGF-2/VEGF Quantikine ELISA Kits (R&D Systems, #DGR00B, #D8000C, #DFB50, #DVE00); mouse VEGF Quantikine ELISA kit (R&D Systems #MMV00); human CD63/TSG101 ELISA kits (LSBio, #LS-F11093, #LS-F8581); HRP-conjugated anti-human IgG (Fc specific) (Sigma-Aldrich, #A0170, 1:5000 dilution)

5) Other antibodies: anti-CD31 (for IHC, Abcam, #ab28364, 1:50 dilution); anti-GFP (for IHC, Abcam, #ab5450, 1:500 dilution); anti-VEGFR2 (for neutralization, R&D Systems, #mab3572, 250ng/mL); normal mouse IgG (Thermo Fisher Scientific, #10400C, 250ng/mL); normal human IgG (Innovative Research, #IR-HU-GF-ED, 5mg/kg)

Validation

All antibodies are well-established and are commonly used and published reagents, with expected results described by the company. No validation was required.

Eukaryotic cell lines

Policy information about cell lines

Cell line source(s)

HUVEC and parental ES2, HCT116, 786-0, CHO-K1 and pgsD-677 cell lines were purchased from ATCC.

Authentication

Cell lines that were used in this study were authenticated by Short Tandem Repeat analysis that was performed by the University of Texas MD Anderson Characterized Cell Line Core Facility.

Mycoplasma contamination

Cell lines that were used in this study were tested for mycoplasma contamination and they were free of mycoplasma contamination.

Commonly misidentified lines
(See ICLAC register)

No commonly misidentified cell lines were used.

Animals and other organisms

Policy information about studies involving animals; ARRIVE guidelines recommended for reporting animal research

Laboratory animals

Four-week-old female nude mice (purchased from University of Texas MD Anderson Cancer Center animal facility) were used in this study. These mice were housed in the SPF barrier facility at the University of Texas MD Anderson Cancer Center.

Wild animals

No wild animals were used.

Field-collected samples

No field-collected samples were used.

Ethics oversight

All animal studies were conducted in accordance with protocols approved by the University of Texas MD Anderson Cancer Center Institutional Animal Care and Use Committee.

Note that full information on the approval of the study protocol must also be provided in the manuscript.

Human research participants

Policy information about studies involving human research participants

Population characteristics

This study used archived, residual clinical specimens of human tissue and biological fluids that were not necessary for diagnosis and that received full informed consent from all human subjects. Patient cohorts are described in the Methods and in previous reports cited in the References.

Recruitment

No human subjects were recruited for this study.

Ethics oversight

Studies using human clinical specimens were approved by the Institutional Research Board of the University of Texas MD Anderson Cancer Center and the Institutional Research Board of the University of Chicago.

Note that full information on the approval of the study protocol must also be provided in the manuscript.

Flow Cytometry

Plots

Confirm that:

- [x] The axis labels state the marker and fluorochrome used (e.g. CD4-FITC).
- [x] The axis scales are clearly visible. Include numbers along axes only for bottom left plot of group (a 'group' is an analysis of identical markers).
- [x] All plots are contour plots with outliers or pseudocolor plots.
- [x] A numerical value for number of cells or percentage (with statistics) is provided.

Methodology

Sample preparation

To analyze uptake of sEVs, sEVs were labeled with PKH26 red fluorescent cell linker dye (Sigma-Aldrich). HUVEC (100,000 cells) were incubated with PKH26-labeled sEVs (100 microgram/mL) at 37°C for times indicated in the figure legends. Thereafter, HUVEC were harvested by treatment with trypsin, washed with PBS and then resuspended in 1% BSA in PBS.

To analyze sEV surface proteins by flow cytometric analysis of binding to antibody-coupled microbeads, sEVs (100 micrograms) were incubated with antibody-coupled Exo-Flow FACS microbeads (640,000 beads of 9.1 micrometer in diameter, System Biosciences) at 4°C for 16 h with rotation. Details of antibodies that were coupled to microbeads and concentrations used are described in the figure legends, methods and reporting summary. Thereafter, microbeads were washed with 1% BSA in PBS , stained with exo-FITC dye to label sEV membrane (Systems Biosciences) and then resuspended in fresh 1% BSA in PBS.

To analyze sEV surface proteins by flow cytometric analysis of direct staining of sEVs, settings were optimized by using the Nanobead calibration kit (50nm and 100nm diameter fluorescent beads) and submicron bead calibration kit (200nm and 500nm diameter beads) both purchased from Bangs Laboratories. sEVs (100 micrograms) were incubated with PE-conjugated VEGF Ab or isotype control (1:100 dilution) in combination with PE/Cy7-conjugated CD63 Ab or isotype control (1:100 dilution) at room temperature for 30 min. Following incubation, samples were diluted in PBS and acquired.

To analyze sEV surface proteins following enzymatic digestion, PKH67-labeled sEVs (100 micrograms) were suspended in digestion buffer (DMEM supplemented with 0.5% BSA and 20 mM HEPES-HCl (pH 7.4)) and incubated with heparinase I and III blend (1 mU/mL) or with chondroitinase ABC (50 mU/mL) at 37°C for 3 h. Fresh enzyme was added and samples incubated for a further 16 h. sEVs were then extensively washed with PBS and concentrated by using Centricon® filter units. Thereafter, sEVs were incubated with antibody-coupled microbeads at 4°C for 16 h with rotation as described above, followed by washing with 1% BSA in PBS and resuspension in fresh 1% BSA in PBS.

To analyze intracellular VEGF, cancer cells (1,000,000 cells) were incubated at 37°C for 6 h with the addition of brefeldin A (BioLegend) (5 microgram/mL) to block protein secretion. Thereafter, cells were harvested by treatment with trypsin and washed with PBS. Cells were then fixed with 1% paraformaldehyde in PBS at 4°C for 20 min and then permeabilized in 0.1% saponin at room temperature for 15 min. Following washing with 1% BSA in PBS, cells were incubated with bevacizumab or VEGFR1/R2-Fc (25 microgram/mL) at 4°C for 30 min and then washed. Cells were stained with PerCP-conjugated anti-human IgG (Fc specific) (1:100 dilution) at 4°C for 30 min, then washed and fixed in 4% paraformaldehyde in PBS.

To confirm that bevacizumab was coupled to microbeads, bevacizumab-coupled microbeads were incubated with Ab to bevacizumab (1:100 dilution) at 4°C for 30 min and then washed with 1% BSA in PBS. Thereafter, microbeads were incubated with PerCP-conjugated anti-mouse IgG (1:100 dilution) at 4°C for 30 min, washed and then acquired.

All of the above samples were protected from exposure to light during preparation.

Instrument

Data was collected using a BD FACSCalibur and a BD FACSCanto II (BD Biosciences)

Software

CellQuest Pro , FACS Diva software (BD Biosciences) and FlowJo software (FlowJo LLC) were used to collect and analyze flow cytometry data

Cell population abundance

N/A

Gating strategy

FSC and SSC gating was used to select the population of live HUVEC cells within which PKH26 fluorescence was analyzed to determine uptake of sEVs. FSC and SSC gating was also used to select the population of permeabilized cancer cells within which PerCP fluorescence was analyzed to determine intracellular staining of VEGF. FSC and SSC gating was used to select the population of singlet antibody-coupled microbeads within which exo-FITC or PKH67 fluorescence was analyzed to determine the binding of sEVs, or within which PerCP fluorescence was analyzed to detect bevacizumab. Furthermore, FSC and SSC gating was used to select the population of 100nm diameter sEVs within which PE and PE/Cy7 fluorescence was analyzed to determine surface expression of VEGF and CD63 on sEVs. A minimum of 10,000 gated events was analyzed for each sample.

- [x] Tick this box to confirm that a figure exemplifying the gating strategy is provided in the Supplementary Information.
